# Gut microbiota dysbiosis and bacterial community assembly associated with cholesterol gallstones in large-scale study

**DOI:** 10.1186/1471-2164-14-669

**Published:** 2013-10-01

**Authors:** Tao Wu, Zhigang Zhang, Bin Liu, Dezhi Hou, Yun Liang, Jie Zhang, Peng Shi

**Affiliations:** 1 Department of Hepatobiliary Surgery, The First Affiliated Hospital of Kunming Medical University, Kunming 650032, China; 2 State Key Laboratory of Genetic Resources and Evolution, Laboratory of Evolutionary & Functional Genomics, Kunming Institute of Zoology, Chinese Academy of Sciences, Kunming 650223, Yunnan, China; 3 Department of Hepatobiliary Surgery, The Second Clinical College of Kunming Medical University, The Second Affiliated Hospital of Kunming Medical University, Kunming 650101, Yunnan, China

**Keywords:** Gut microbiota dysbiosis, Cholesterol gallstone, Bile, Bacterial colonization, Pyrosequencing

## Abstract

**Background:**

Elucidating gut microbiota among gallstone patients as well as the complex bacterial colonization of cholesterol gallstones may help in both the prediction and subsequent lowered risk of cholelithiasis. To this end, we studied the composition of bacterial communities of gut, bile, and gallstones from 29 gallstone patients as well as the gut of 38 normal individuals, examining and analyzing some 299, 217 bacterial 16S rRNA gene sequences from 120 samples.

**Results:**

First, as compared with normal individuals, in gallstone patients there were significant (*P* < 0.001) increases of gut bacterial phylum Proteobacteria and decreases of three gut bacterial genera, *Faecalibacterium*, *Lachnospira*, and *Roseburia*. Second, about 70% of gut bacterial operational taxonomic units (OTUs) from gallstone patients were detectable in the biliary tract and bacteria diversity of biliary tract was significantly (*P* < 0.001) higher than that of gut. Third, analysis of the biliary tract core microbiome (represented by 106 bacteria OTUs) among gallstone patients showed that 33.96% (36/106) of constituents can be matched to known bacterial species (15 of which have publicly available genomes). A genome-wide search of *MDR*, *BSH*, *bG*, and *phL* genes purpotedly associated with the formation of cholesterol gallstones showed that all 15 species with known genomes (e.g., *Propionibacterium acnes*, *Bacteroides vulgates*, and *Pseudomonas putida*) contained at least contained one of the four genes. This finding could potentially provide underlying information needed to explain the association between biliary tract microbiota and the formation of cholesterol gallstones.

**Conclusions:**

To the best of our knowledge, this is the first study to discover gut microbiota dysbiosis among gallstone patients, the presence of which may be a key contributor to the complex bacteria community assembly linked with the presence of cholesterol gallstones. Likewise, this study also provides the first large-scale glimpse of biliary tract microbiota potentially associated with cholesterol gallstones. Such a characterization of the biliary tract core microbiome has potentially important biological and medical implications regarding the role of bacteria in the formation cholesterol gallstones.

## Background

With a prevalence among adults somewhere between 10–15% in Europe and the USA, gallstones are the most common gastrointestinal disorder requiring hospitalization in the West [1]. Most literature to date points to abnormal metabolism and secretion of cholesterol and bile acids as the primary pathophysiological defect in the formation of gallstones [2,3], but rather intriguingly, gut microbiota enables the regulation of bile acid metabolism by reducing bile acid pool size and composition [4]. This role indicates that gut microbiota is likely to be at least partially associated with the formation of gallstones [5]. Similarly, the presence of gut microbiota members in lithogenic bile [6,7] could be an indication of increased intestinal permeability during biliary obstruction [8], which then may contributing to increased inflammatory response and stone formation [5]. Such an association is not illogical— recent studies continue to show that human-associated microbial communities are linked with a variety of diseases, e.g., cirrhosis with minimal hepatic encephalopathy, nonalcoholic fatty liver disease, obesity, type 2 diabetes, and Crohn’s disease (CD) [9-13]. However, little is known about whether gut microbiota dysbiosis is connected with cholelithiasis or could be correlated to the complex bacterial colonization associated with the formation of gallstone. Consequently, a better understanding of bacterial communities in both gut and biliary tract of gallstone patients is crucial in developing a greater understanding of the connection between disease and the microflora communities in the body and then developing strategies to promote digestive tract health.

Bacteria may be one of major factors at play in the pathogenesis of gallstones. MAKI [14] first proposed that bacterial infection may play a key role in the pathogenesis of pigment gallstones, and since then, studies continue to highlight the association of bacterial infection with the formation of gallstones, [1,15-23], especially among cholesterol stones, which make up nearly 80% of all cases of gallstones in the USA. Although debate continues regarding the role of bacteria in the pathogenesis of cholesterol gallstone [24,25], outright denial of the presence of bacteria (e.g., *Pseudomonas aeruginosa*, *Entercoccus spp.*, *Escherichia coli*, and *H. pylori*) in cholesterol gallstones is simply incorrect [21-23], especially as living bacteria can be cultured from cholesterol gallstones [20].

An increasing number of molecular studies continue to indicate that, to some extent, an underlying association exists between bacterial infection and gallstone formation. For example, multidrug-resistance (MDR) efflux pump proteins expressed by bacteria can produce bile resistance that allows bacteria to survive in certain ecological niches alongside bile salts [26-30]. To date, there are five known families of MDR efflux-pump proteins: the ABC super-family, and the MFS, MATE, SMR, and RND families [26]. Of these, the bacteria *Campylobacter jejuni* efflux pump CmeABC has been shown to confer resistance to bile [31,32]. Other potential bacterial factors associated with the formation of gallstones have also been identified, such as *β*-glucuronidase (bG), phospholipases (phL), and bile acid hydrolases (BSH) [33,34].

Increasing evidences may be implicating the role of bacteria role in the formation of cholesterol gallstones. For example, enterohepatic helicobacters can promote formation of cholesterol gallstones in vivo [15]. Urease-positive *Helicobacter* spp can precipitate calcium salts in vitro [35]. Monoinfection with ureasepositive helicobacters or coinfection with at least one urease-positive helicobacter is necessary to induce cholesterol gallstones [15,35]. Interestingly, mice coinfected with several bacteria developed cholesterol gallstones at higher prevalence of 80% than that infected with single bacteria at prevalence of below 40% [15]. These findings indicated that bacterial community assembly might be more important than single species in the formation of cholesterol gallstones. Therefore, to better understand the role bacteria play in the pathogenesis of cholesterol gallstones, large-scale discovery of microbial composition and diversity associated with cholesterol gallstones is critical first step.

Previous studies yielded little insight regarding gallstone-associated microbiota, due largely to the insensitivity of cultivation [18,36] and the relative paucity of sequence analysis of sequenced gene fragments based on molecular finger-printing methods [37] and cloned microbial small-subunit ribosomal RNA genes [16S ribosomal DNA (rDNA)] [17,19,22]. The unfortunate outcome of these shortcomings is that despite substantial progress in a number of fields, we still lack an integrated view of the composition, structure, and origin of gallstone-associated bacterial community. Recent advances in the technology (e.g., a cost-effective method based on culture-independent 454 pyrosequencing of the bacterial 16S rRNA gene) used to identify and to analyze components of the microbiome have substantially improved our understanding of the microbial communities associated with this human disease [38]. In this study, using 454-barcoded- pyrosequencing, we undertook a large-scale molecular analysis of 16S rDNA sequences in order to gain a clearer picture of four crucial issues: 1) the disorder of gut microbiota with cholelithiasis; 2) the structure and component of biliary tract microbial communities in patients with cholesterol gallstones; 3) the relation between bacterial communities in both the gut and biliary tract; 4) the characteristics of biliary tract core microbiome and its potential connection in the formation of cholesterol gallstones.

## Results

### Sample and data collection

In total, we collected samples from 29 patients with gallbladder stones (hereafter referred to as patients) and 38 healthy subjects (hereafter referred to as subjects) (see Methods for detailed information). From the 29 gallbladder-stone patients we obtained samples of bile, gallstones and feces: 26 bile samples, 27 gallstones (2 with 50% cholesterol content, 24 with 70-90%, and 1 over 95%), and 29 fecal samples. According to the definition of cholesterol stones with the main composition of 50-90% cholesterol contents recommended by Marschall et al. [39], all collected 27 gallstones are properly classified as cholesterol gallstones. More strictly, however, using 70% cholesterol content cutoff proposed by Swidsinski et al. [25] and Portincasa et al. [1], 92.59% of our sampled gallstones are cholesterol gallstones. We further collected 38 normal human feces samples (data of 27 samples was collected in one of our recent studies [13]), yielding a total of 120 samples, which were used in our molecular analysis of the bacterial 16S rRNA genes.

For each sample, variable regions (V1-V2) of the bacterial 16S ribosomal RNA (rRNA) gene was amplified vi polymerase chain reaction (PCR) using a primer set with a unique 10-nt barcode. From all the bile, stone and fecal samples, we obtained a dataset consisting of 299, 217 high-quality 16S rRNA gene sequences with an average of 2493 ± 127 (S.E.) (n = 120) sequences per sample (Table 1). From the dataset, we identified a total of 4637 operational taxonomic units (OTUs) based on the conventional criterion of 97% sequence similarity (equal to species level), with 3696 OTUs in bile, 3456 OTUs in gallstones (we detected bacteria irrespective of cholesterol content in all gallstones), 1772 OTUs in patient feces, and 1497 OTUs in normal feces, [40], with an average of 321 ± 29 [Good’s Coverage [41]: 92.8 ± 0.6%] per sample (n = 120) (Table 1). The taxonomic summary of microbial components from all samples (Additional file 1: Figure S1) yielded a total of 20 different bacterial phyla across all samples, with 20 present in gallstones, 19 in bile, 13 in patient feces, 14 in normal feces, with the most dominant being the phylum Firmicutes in bile, gallstone, and feces.

**Table 1:** 454 Data summary

	**Normal feces**	**Patient feces**	**Patient bile**	**Patient stone**	**Totally**
	**(N = 38)**	**(N = 29)**	**(N = 26 )**	**(N = 27)**	**(N = 120)**
Total sequences	77061	64360	81661	76135	299, 217
Sequences per sample	2028 ± 186	2219 ± 175	3141 ± 310	2820 ± 313	2493 ± 127
Total OTUs	1, 497	1, 772	3, 696	3, 456	4, 637
OTU number per sample^***^	178 ± 13	167 ± 26	593 ± 73	470 ± 73	321 ± 29
Singletons^***^	81 ± 5	78 ± 15	240 ± 44	241 ± 39	166 ± 16
Good coverage (%)	95.1 ± 0.4	96.1 ± 0.5	89.1 ± 1.6	89.7 ± 1.7	92.8 ± 0.6
Shannon diversity index^***^	4.97 ± 0.15	4.59 ± 0.21	6.84 ± 0.46	6.25 ± 0.44	5.57 ± 0.18
Chao1^a,***^	299.8 ± 20.3	296.9 ± 51.3	1075.9 ± 147.1	847.3 ± 131.1	590.4 ± 54.5
PD - whole tree^a,***^	10.4 ± 0.7	11.4 ± 1.4	33.1 ± 3.0	28.3 ± 3.1	19.6 ± 1.4

^***^ significant difference of *P* < 0.001, based on Kruskal-Wallis one way analysis of variance on ranks. ^a^Phylogenetic-based alpha diversity measure. PD, Phylogenetic Diversity.

Note: Significant difference was observed between the gut and biliary tract but not within gut or biliary tract, indicating higher microbial diversity in the biliary tract than in the gut.

### Comparative metagenomic analysis between and within gut and biliary tract

Comparative metagenomic analysis uncovered a significantly higher difference (*P* < 0.001) of microbial diversity in the biliary tract as compared with the gut (richness given by OTUs and singletons as well as evenness by Shannon index, Chao1, and PD-whole tree), though not within the biliary tract (bile vs. gallstone) (Table 1). This level of microbial community diversity found in biliary tract of gallstone patients was unprecedented in our current knowledge of the healthy human body, as only the human skin harbors a more diverse microbial community [42].

**Figure 1:**
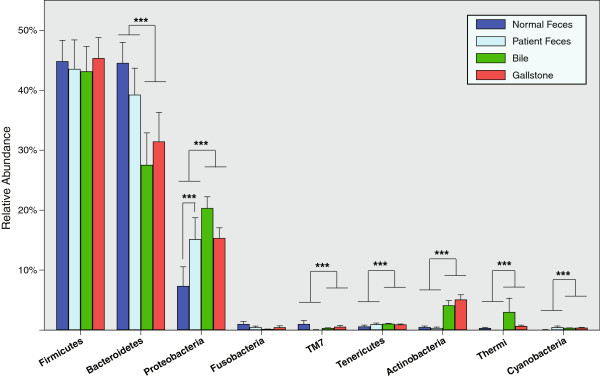
**Phyla level comparisons of microbial components between the gut and biliary tract.** Only top nine phyla shown. ***P < 0.001.

**Figure 2:**
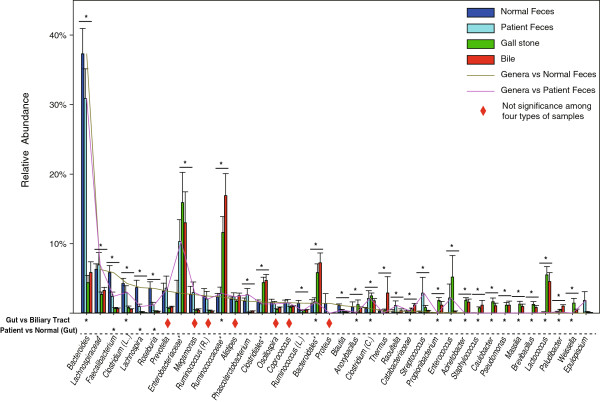
**Genera level comparisons of microbial components between gut and biliary tract.** In total, 38 genera distributions are shown, including those genera of each type of sample with relative abundance of at least 0.01%, respectively. Taxons marked by stars were unclassified. *C.*, *Clostridiaceae* family. *L.*, *Lachnospiraceae* family. *R.*, *Ruminococcaceae* family. *P < 0.05.

At the phylum level (Figure 1), we found no significant differences between and within gut and biliary tract in either the most dominant phylum Firmicutes or the rare phylum Fusobacteria. Conversely, when compared with gut, we found significantly (*P* < 0.001) decreased levels of the bacterial phylum Bacteroidetes in the biliary tract. We further observed significantly (*P* < 0.001) increase of six bacterial phyla in the biliary tract, including Proteobacteria, TM7, Tenericutes, Actinobacteria, Thermi, and Cyanobacteria. Within the gut, the relative abundance of the bacterial phylum Proteobacteria among patients were significantly (*P* < 0.001) higher than that in normal. Within biliary tract, we observed no difference of those bacterial phyla between bile and gallstones. At the genus level (Figure 2), 7 bacteria genera showed no significant differences between and within the gut and biliary tract. Compared with the gut genus composition, in the biliary tract, there was a significant (*P* < 0.001) decrease of 2 bacterial genera accompanied by an increase in 16 other bacterial genera Within the gut, the relative abundance of 3 bacterial genera (*Faecalibacterium*, *Lachnospira*, and *Roseburia*) were significantly (*P* < 0.001) lower in patients as compared with healthy subjects.

**Figure 3:**
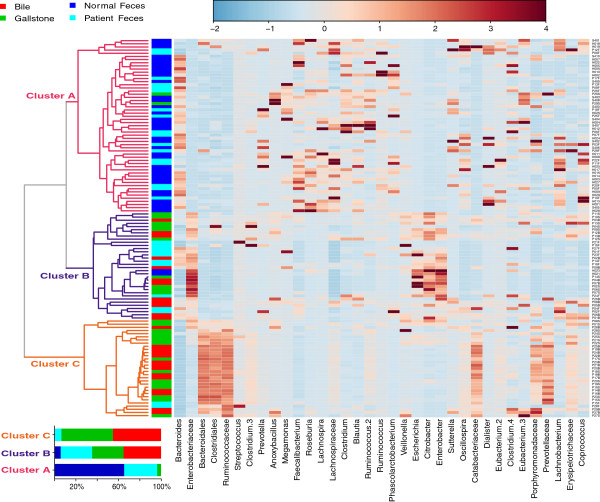
**Microbiota analysis of separates samples based upon where they live in the community.** Hierarchical average clustering based on the Spearman/Pearson correlation coefficients of the proportion of all taxons, filtered for taxonomic variables with over 50% percent zeroes. Non-informative variables are characterized using the coefficient of variation (mean/standard deviation). Data normalization between samples was conducted by Sum, and those between taxons Pareto Scaling (mean-centered and divided by the square root of standard deviation of each variable). In total, 35 taxonomic features are shown in the heatmap, with statistical significance among four types of samples based on *T*-test/ANOVA analyses.

**Figure 4:**
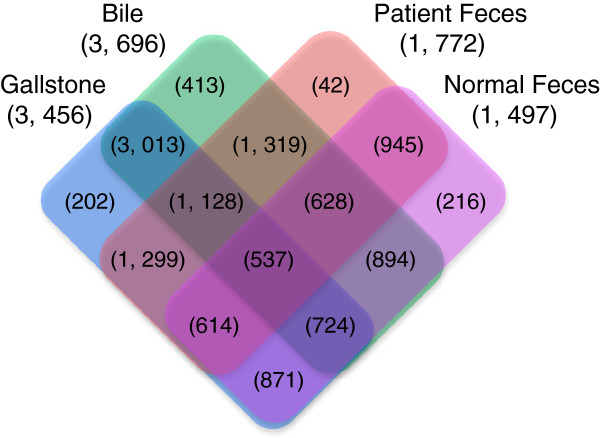
**Quantitative comparisons of bacterial OTUs between the gut and biliary tract.** Numbers in brackets denote OTU number discovered from each of four different types of samples, corresponding to four different colored boxes. Number in brackets located at the colored boxes denotes shared OTUs (at overlapped boxes) across either the four types of samples or those unique to a given sample type.

Our results further found that 120 samples could be divided into three distinct clusters (A-C) (Figure 3). Gut microbiota was predominant within cluster A, biliary tract microbiota within cluster C, and the crossover between gut (in particular among patients) and the biliary tract within cluster B. A similar pattern was also discovered via PLS-DA plot (Additional file 2: Figure S2) and weighted & unweighted UniFrac PCoA plot (Additional file 3: Figure S3) based on microbiota analysis. We further found that over 85% of bacterial OTUs were shared in both bile and gallstones (Figure 4), but only 60% of gut bacterial OTUs were shared by patients and healthy subjects. Around 70% of gut bacterial OTUs from patients and 40% OTUs from healthy subjects were found in the biliary tract.

### Characteristics of the biliary tract core microbiome in gallstone patients

**Figure 5:**
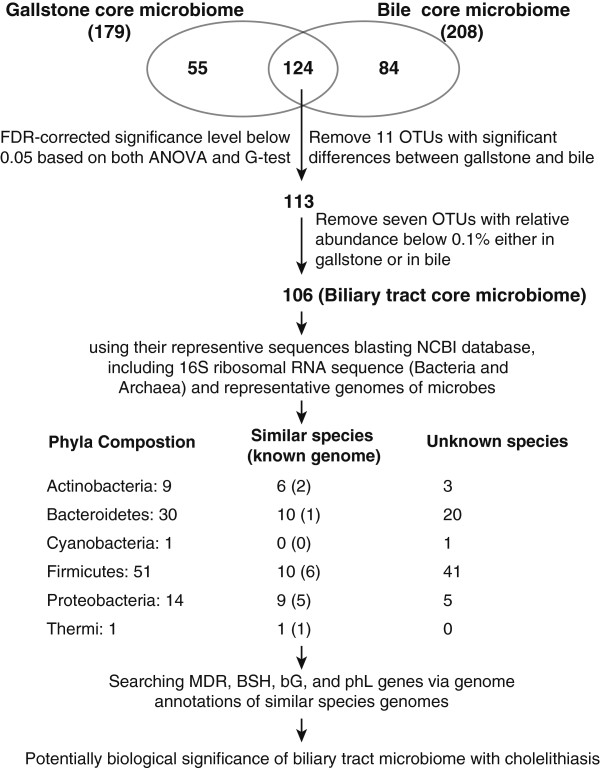
Pipeline for identifying biliary tract core microbiome and potential microbial species associated with the presence of gallstones.

We identified a core microbiome of both bile and gallstones by using a general rule [43] of including a bacterial OTU (species) if it was shared by at least half of the samples in a given group (i.e., either patient bile or gallstones). The bile core microbiome consisted of 208 bacterial OTUs while the gallstone core microbiome consisted of 179 bacterial OTUs. Based on those datasets, we identified a biliary tract core microbiome for gallstone patients, which includes 106 bacterial OTUs belonging to 6 bacterial phyla. Results from using the QIIME pipeline revealed 33.96% (36/106) of the constituent members of the biliary tract core microbiome could be matched to known bacterial species (Figure 5), according to at least 97% identity (Additional file 4: Table S1).

Though the results of our previously mentioned analysis was intriguing, further characterizing the functional features of the biliary tract microbiome via a culture approach proved quite difficult. To overcome this defect, we found relative clues by searching function gene contents of similar species with available sequenced genomes. Previous reports have confirmed the feasibility of this study [44]. Pipline analysis results (Figure 5) also showed that 15 of the 36-matched bacterial species have publicly available genomes. Based on reference genomes of those 15 bacterial species, we searched for the presence or absence of four genes potentially associated with the formation of gallstones: *MDR*, *BSH*, *bG*, and *phL*. The results (Additional file 5: Table S2) found 15 species that contained at least contained one of the four genes, 20% of which had all four genes, 40% had three of them, and 40% had two of them. The *MDR* gene was the most abundant in all 15 species (totally identified gene number ranging from 16 to 59), while the *BSH* gene was discovered in 5 species (33%) (ranging from 1 to 3), *bG* gene in 7 species (47%) (the most 24 members in *Bacteroides vulgates*, others with 1 to 3), and the *phL* gene in 13 species (87%) (ranging from 1 to 5).

## Discussion

The results of this study, which used a targeted amplicon sequencing approach, demonstrates an underlying gut microbiota dysbiosis present with cholelithiasis and that biliary tract microbiota has the potential to promote the formation of gallstones, in particular cholesterol gallstones. This is the first study to use a 454-based pyrosequencing of bacterial 16S rDNAs to clarify the composition of both gut and biliary tract microbiota with cholelithiasis. While methods such as ANOVA, hierarchical clustering via heatmap, and UniFrac-based PCoA analysis can identify differences of microbial components and diversity both between and within the gut and biliary tract, culture-independent 454 pyrosequencing of 16S rRNA genes provides unprecedented insight into gallstone-associated microbiota. Two of our recent studies demonstrated an association between gut microbiota dysbiosis and human diseases, e.g., colorectal cancer [45] and cirrhosis with minimal hepatic encephalopathy [13], validating the relevant methods used in this study.

Our results discovered significant changes of gut microbial components between gallstone patients and healthy subjects. Within the gut of patients, there exists an overgrowth of the bacterial phylum Proteobacteria which generally includes a wide variety of pathogens such as *Escherichia*, *Salmonella*, *Vibrio*, and *Helicobacter*. These pathogens have variously been linked with many gastrointestinal tract (GIT) diseases. A classical case is that a major pathogenic *Escherichia coli* O104:H4 outbreak occurred in central Europe during late spring of 2011 [46]. Furthermore, three gut bacterial genera—*Faecalibacterium*, *Lachnospira*, and *Roseburia—*were significantly reduced in gallstone patients. The decrease of *F. prausnitzii* has been closely related to gut dysbiosis in patients with CD [47], but *F. prausnitzii* were significantly increased after specific oligosaccharide (e.g., inulin–oligofructose) consumption [48]. Meanwhile, the gut bacteria *R. inulinivorans* are inulin and starch consumers [49], while *Lachnospira* are pectin degraders [50]. The previous experimental studies showed that via the consumption of oligosaccharide, *R. inulinivorans* and *Lachnospira* may selectively stimulate the growth of potentially beneficial gut bacteria such as *F. prausnitzii*. The co-occurrence of those three gut bacteria likely has potential effects on gut health of gallstone patients, though what those effects may be is not currently known. Ultimately, the co-occurring gut microbiota dysbiosis demonstrated in this study is indicative of unpredictable GIT health risks among gallstone patients that remain largely unknown.

This study supports mounting evidence that culture-independent 454-based 16S rDNA sequencing is necessary as a diagnostic tool in gallstone-associated bacterial infection, because it provides more detailed information than cultivation or other molecular finger-printing methods currently employed [37] and sequence analysis of cloned microbial 16S rDNA [17,19,21]. Our results likewise demonstrated less than 5% of 3456 bacterial OTUs identified in gallstones have been reported in previous studies [14,15,17-19,21-23,25,51-59]. Conservatively, only 34% (36) of 106 core bacteria OTUs in the biliary tract of gallstone patient are known to date, such as *Escherichia coli*[60]. Surprisingly though, around 70% of gut bacterial OTUs from gallstone patients were detectable in the biliary tract. This raises a possibility that biliary tract microbiota could be originated from the gut. Further evidence is obviously needed to explore this possibility, but nonetheless it is an intriguing prospect.

Interestingly, members of *Lactococcus,* which are generally used as probiotics in human gastrointestinal tract, [61] were enriched in biliary tract of those patients with cholelithiasis (Figure 2). However, unlike the bile salt hydrolytic activities of *Lactobacilli*[62], the underlying physiological role of *Lactococcus* in cholelithiasis remains unclear. Such unknowns are compounded by significant inter-subject variations of the biliary tract microbiota were also observed (Additional file 6: Figure S4). For example, the highest abundance of *Firmicutes* phylum in one gallstone patient was 93.30% and the lowest was 1.17% in another; a similar result was also seen in bile with a high of 55.10% and low of 0.08%. High person-to-person variation in biliary tract microbiota could be related to a number of factors—host diets, lifestyles, genotypes, disease status, etc.

Aside from interpersonal variation, there are several potential factors that may affect the colonization or survival of bacteria in biliary tract of gallstone patients. First, MDR efflux-pump proteins expressed by bacteria can produce bile resistance, allowing bacteria to survive in their ecological niche [26-30], as well as BSH activity [62,63] produced by bacteria that can protect the bacterial cells which produce it from the toxicity of conjugated bile salts. Our results found abundant MDR and BSH genes in the bile tract microbiome, partially supporting the above inference. Second, biopsies taken from the bile ducts of healthy individuals are either free of bacteria or bacteria is quite rare. Though there is not a clear explanation for this based on our existing knowledge, it is possible that a loss of host resistance to microbial colonization in biliary tract may lead to the presence of biliary tract microbiota. Third, developing gallstones play a significant role in gallbladder colonization and carrying of specific bacteria species, such as *Salmonella* species [64], implying that the gallstones themselves may actually aggravate the overgrowth of bacteria in the biliary tract.

Colonization of the biliary tract by varying bacteria may contribute to bile cholesterol super-saturation, which is one of the risk factors in the formation of cholesterol calculus. According to the ternary equilibrium “bile salt-phospholipid-cholesterol” ternary phase diagram [65-68], cholesterol is only slightly soluble in an aqueous media, but is made soluble in bile via mixed micelles by bile salts and phospholipids, mainly phosphatidylcholine, whose concentrations determine the degree of cholesterol saturation. Studies further indicate that two bacterial factors *bG* and *PhL* genes facilitate gallstone formation [34]. The most abundant member of the *PhL* gene in bile, phospholipase A(2), can induce cholesterol gallstone formation by hydrolyzing bile phospholipids into lysolecithin and free fatty acids [69]. The results of the present study showed that both the *bG* and *PhL* genes were abundant in the biliary tract microbiome of gallstone patients (Additional file 5: Table S2), which is partially indicative of increasing risks of cholesterol gallstone that may be a potential consequences of bacterial colonization in human biliary tract. Further research is needed to verify this and some of the other suggestive conclusions, but, despite the preliminary nature of some of our results, the most poignant implication of this study is that prevention and management of bacterial infections in the biliary tract may be a target for lowering the risks of cholesterol gallstones.

## Conclusions

To the best of our knowledge, this is the first study to discover a potential association of the gut microbiota dysbiosis that is present among gallstone patients. Likewise, our characterization of the biliary tract core microbiome provides potentially significant biological implications about both the unexpected diversity of the microbiome among patients with cholesterol gallstones, as well as the probable roles of bacteria in the formation of cholesterol gallstones. Further research on the bile tract microbiome’s functionality will likely complement our findings on biliary tract microbiome and clarify some of the implications that arose from our conclusions. Ultimately, these findings have numerous medical implications for both prevention and therapeutics for cholelithiasis or other relative GIT healthy risks, warranting further follow-up studies that are needed to verify these findings and move forward.

## Methods

### Studied subjects and sample collection

All protocols and procedures of this study conformed to the ethical guidelines outlined in the 1975 Declaration of Helsinki as reflected by *a priori* approval from the First Affiliated Hospital of Kunming Medical University of China. Prior to inclusion in this study, all participants provided written informed consent.

Patients with gallstone disease were diagnosed with cholecystolithiasis via B-mode ultrasonography. In total, 29 definite patients from Kunming, China were used in this study with (49.2 ± 14.3) (SD) average age, (23.7 ± 3.2) body mass index (BMI), and (13:16) male/female ratio, and all patients had occurrences of chronic cholecystitis. No patients indicated they had suffered any diseases of the gastrointestinal tract except gallbladder stones, and none had taken antibiotics or probiotics within the previous three months prior to this study. During open cholecystectomy or laparoscopic cholecystectomy, one stone was removed aseptically from the gall bladder from each patient. Concurrently, gallbladder bile from each patient was extracted using 2 mL sterile needle tubing. Likewise, prior to the operation, feces from all patients were also collected. All samples were placed in cryovials without preservative, immediately snap frozen in liquid nitrogen, and stored at −70°C and then transported on dry ice to the Kunming Institute of Zoology, Chinese Academy of Sciences, for sequencing analysis. All samples were stored in their original tubes at −80°C until further processing.

To discover whether there was any significant alteration to the gut microbiome among cholelithiasis patients with chronic cholecystitis, as a normal gut control we used 38 normal Chinese individuals (data from 27 of these was obtained from our recent study [13]) from Kunming, China with a (40.7 ± 14.5) (SD) average age, (21.9 ± 2.4) body mass index (BMI), and male/female ratio of (7:12) all of whom permanently reside in Kunming . All healthy individuals volunteered to accept free routine health examinations at the First Affiliated Hospital of Kunming Medical University. The health status of the healthy volunteers was self-reported. No volunteers indicated they had suffered any diseases of the gastrointestinal tract or any other metabolic diseases such as obesity, diabetes, and cardiovascular disease. None had been subjected to surgical procedures for several years prior to this study. None had taken antibiotics or probiotics within the previous three months of fecal sample collection. Feces samples from all normal individuals were collected and stored in their original tubes at −80°C until further processing.

### Sample preparation, molecular methods and bioinformatics

One stone (≥1.0 cm diameter) from each patient was quadrisected with a fresh, sterile, disposable steel blade. Interior stone material was then loosened with a fresh surgical blade. DNA was extracted from the inner halves of the gallstones, with blades changed between scrapings. Inert materials (e.g., tubings and swabs) collected during the surgical operations were processed identically, and subjected to polymerase chain reaction (PCR) to detect possible contamination during sample storage and processing.

Following pre-treatment of the stones, an aliquot (Gallstone and feces, 180–220 mg; bile, 200 μl) of each sample was suspended while frozen in a solution containing 200 μl buffer ATL (QIAGEN Kit Buffer for bacterial cell lysis) and 200 μl of a slurry of 0.1-mm-diameter zirconia/silica beads (BioSpec Products, Bartlesville, OK). The mixed sample was then lysed by mechanical disruption with a bead beater (BioSpec Products) for 2 min at 20°C), followed by extraction with a QIAamp DNA Stool Mini Kit (Qiagen) using its protocol for isolation of DNA for pathogen detection. Lysis temperature was increased to 95°C for cells which proved difficult to lyse. DNA from each sample was eluted in a final volume of 200 μl elution buffer and stored at – 20°C. Tubes containing only the QIAamp DNA Stool Mini Kit extraction controls were included throughout the lysis and PCR steps to serve as negative controls.

Amplicons of the 16S rRNA gene V1-V2 region were sequenced on a 454 Genome Sequencer FLX Titanium platform, according to previous study [45]. Sequencing reads were quality filtered, OTU clustered, ChimeraSlayer filtered and further analyzed using the QIIME pipeline [45,70], RDP-classifier [71], and weighted & unweighted UniFrac PCoA analyses [72]. All OTUs found in at least two samples were retained for performing the following further analyses. Both hierarchical clustering and PLS-DA plotting of samples based on microbiota analysis were performed using METAGENassist, a comprehensive web server for comparative metagenomics [73].

### Statistical methods

Statistical analysis was performed using SigmaPlot 12.0 (Systat Software, Inc.) and R software packages. General characteristics were expressed as median and mean or percentages. Multiple samples comparisons were performed using one-way ANOVA (parametric) or Kruskal-Wallis one-way analysis of variance on ranks (non- parametric). Statistical significance was set at *P* < 0.05.

### Quantitation of cholesterol in gallstones

Representative gallstone portions were weighed and extracted overnight in 5 ml of chloroform/methanol (2:1). To calculate the cholesterol percentage by weight of each stone, cholesterol was quantified using the enzymatic CHOD-PAP method [74] on a NanoDrop 2000 Spectrophotometer (Thermo-Fisher Scientific) according to the manufacturer’s protocols.

### Availability of supporting data section

All raw 454 sequence data used in this study was deposited to the NCBI Sequence Read Archive (SRA) (accession number SRA096351) (http://www.ncbi.nlm.nih.gov/sra).

## Abbreviations

bG: *β*-glucuronidase; BSH: Bile acid hydrolases; CD: Crohn’s disease; GIT: Gastrointestinal tract; MDR: Multidrug-resistance efflux pump proteins; OTUs: Operational taxonomic units; phL: Phospholipases; rRNA: Ribosomal RNA.

## Competing interests

The authors declare that they have no competing interest.

## Authors’ contributions

TW and ZZ performed the research, analyzed data, and wrote the manuscript; BL, DH, and YL performed research; PS and JZ conceived and supervised the study and revised the manuscript. All authors read and approved the final manuscript.

## Supplementary Material

Additional file 1: Figure S1 Phylum composition of varying samples. ***A***, top three phyla and other rare phyla. ***B***, all 16 rare phyla. Click here for file

Additional file 2: Figure S2 PLS-DA plot of microbial communities present in bile, gallstones, and the gut. Click here for file

Additional file 3: Figure S3 Sample clustering from the gut and biliary tract based on a Unifrac PCoA analysis of bacterial 16S rRNA-derived OTUs. Click here for file

Additional file 4: Table S1 Potentially significant bacteria species associated with the presence of gallstones. Click here for file

Additional file 5: Table S2 Identification of bacterial factors facilitating gallstone formation from 15 similar bacteria species with known genomes. Click here for file

Additional file 6: Figure S4 Inter-subject variations of the top four bacteria phyla present in bile and gallstones. Click here for file
