# Supplementary figures and images for: Gut microbiota dysbiosis and bacterial community assembly associated with cholesterol gallstones in large-scale study
Source: BMC Genomics. 2013 Oct 1;14:669. doi: 10.1186/1471-2164-14-669 (PMC3851472; doi:10.1186/1471-2164-14-669)

**A**

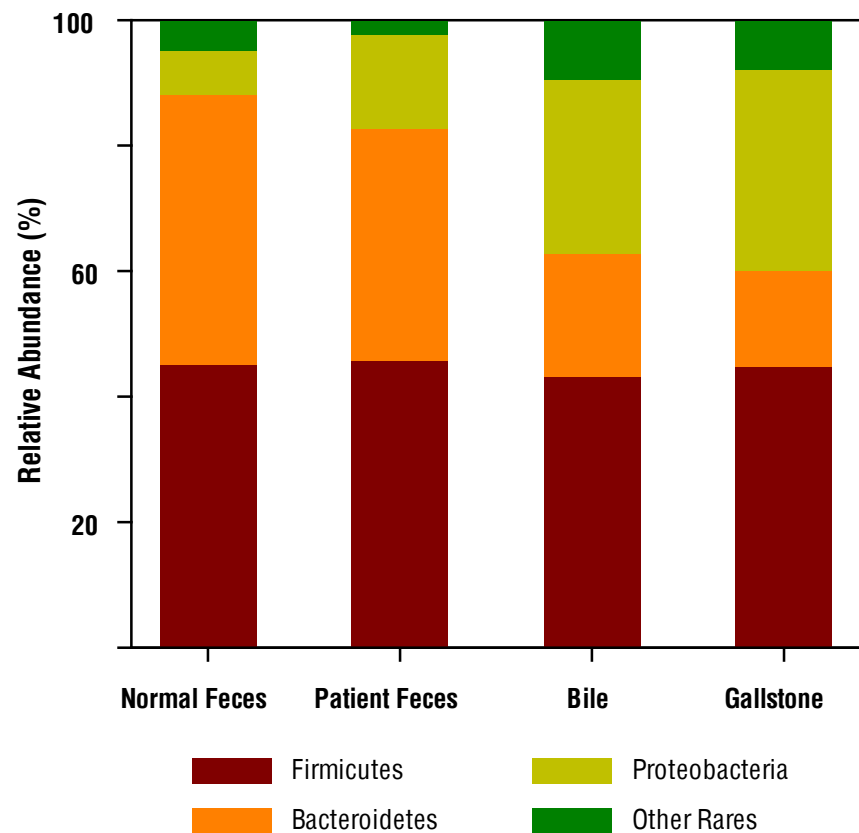

# B

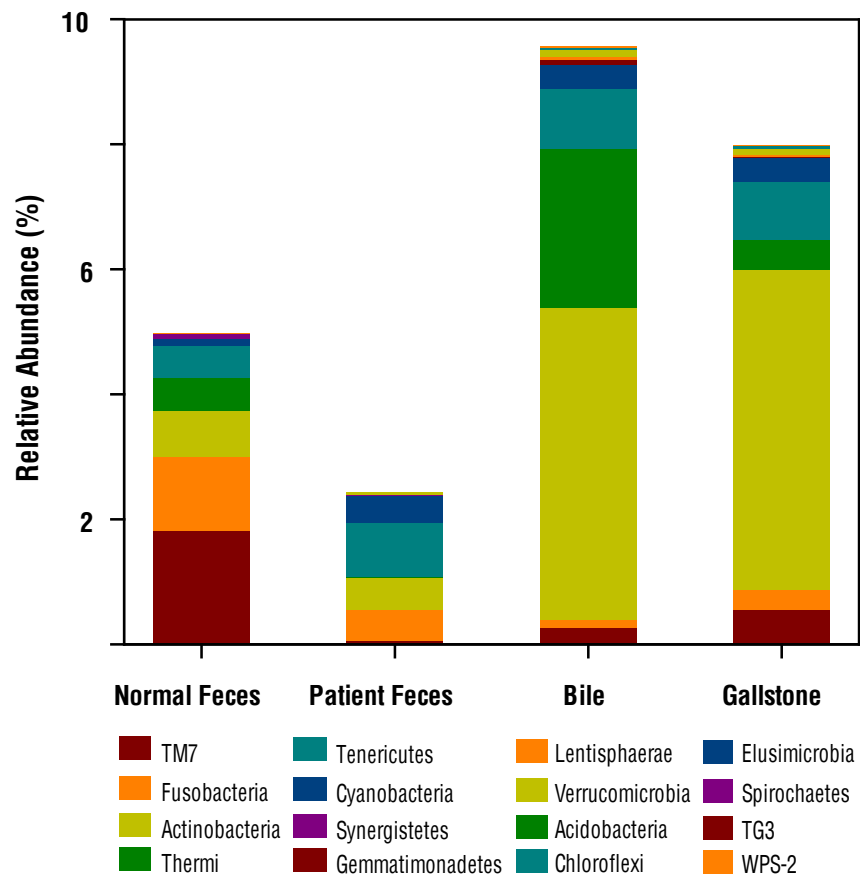

Supplement: Additional file 1: Figure S1 — Phylum composition of varying samples. A, top three phyla and other rare phyla. B, all 16 rare phyla. [file 1471-2164-14-669-S1.pdf]

**Figure S2**

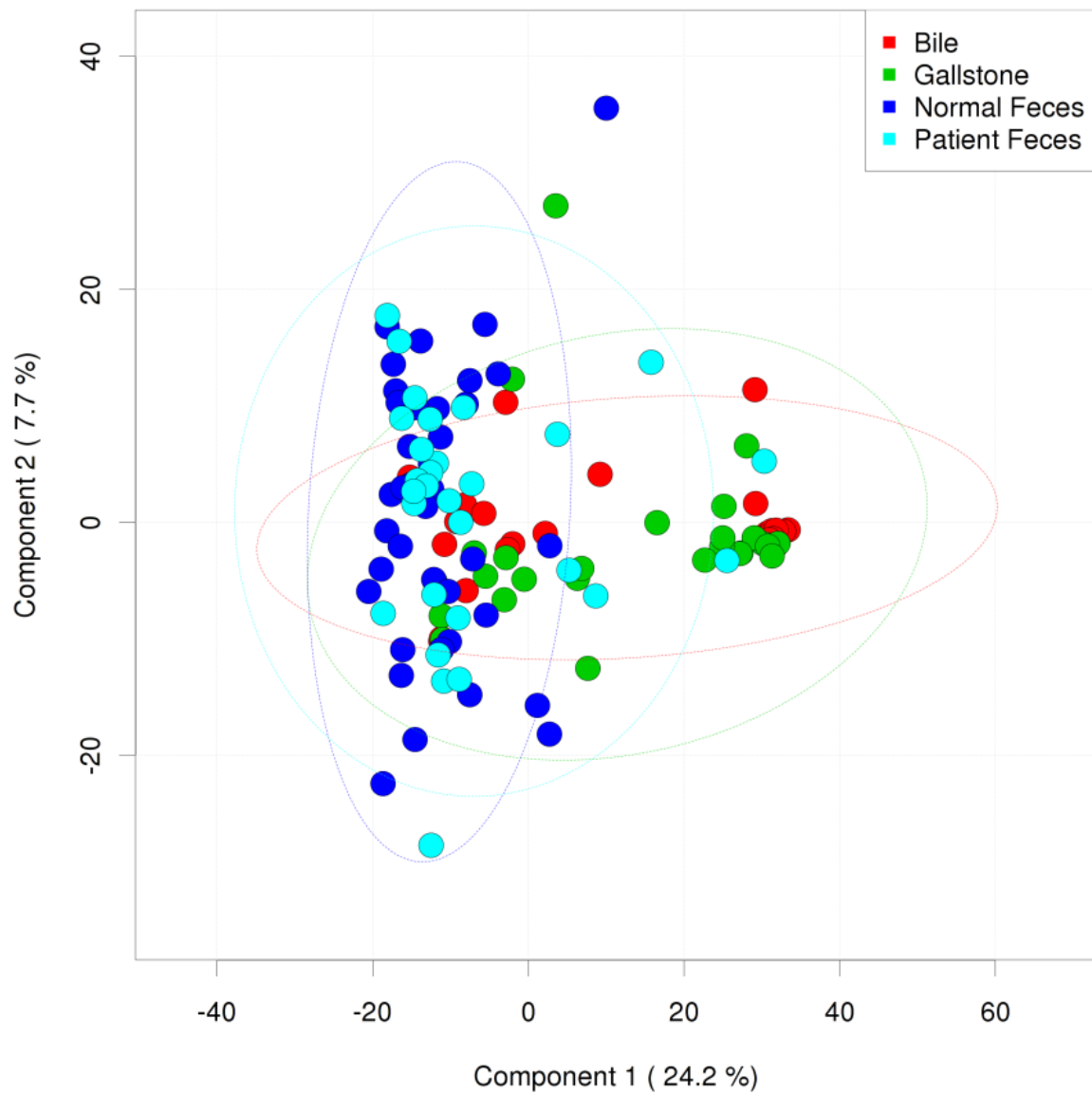

Supplement: Additional file 2: Figure S2 — PLS-DA plot of microbial communities present in bile, gallstones, and the gut. [file 1471-2164-14-669-S2.pdf]

**Figure S3**

**A (unweighted UiFrac PCoA )**

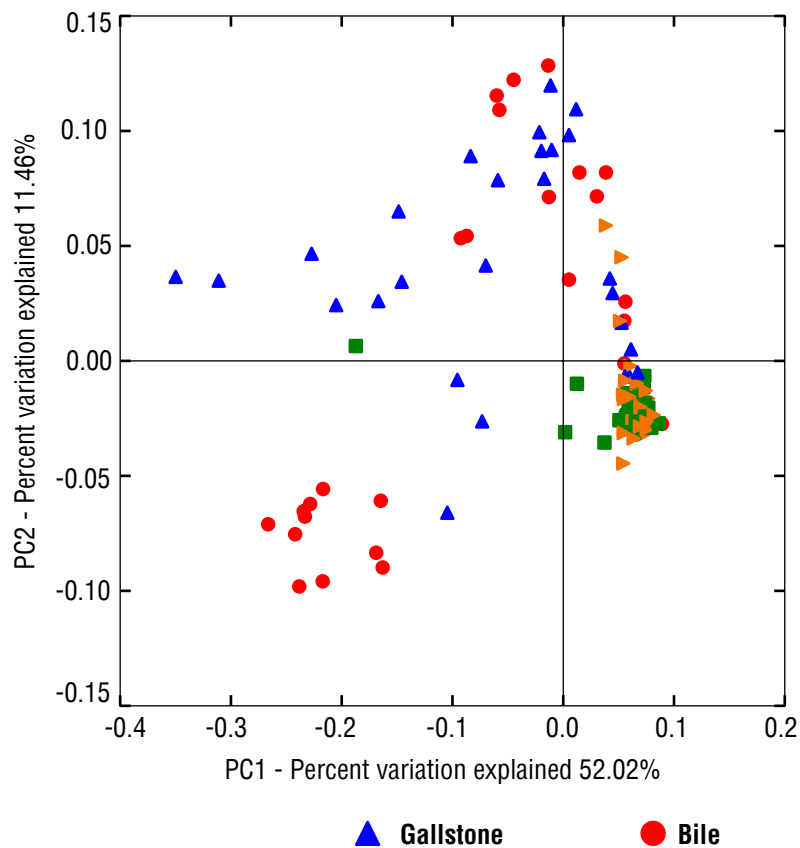

**B (weighted UiFrac PCoA )**

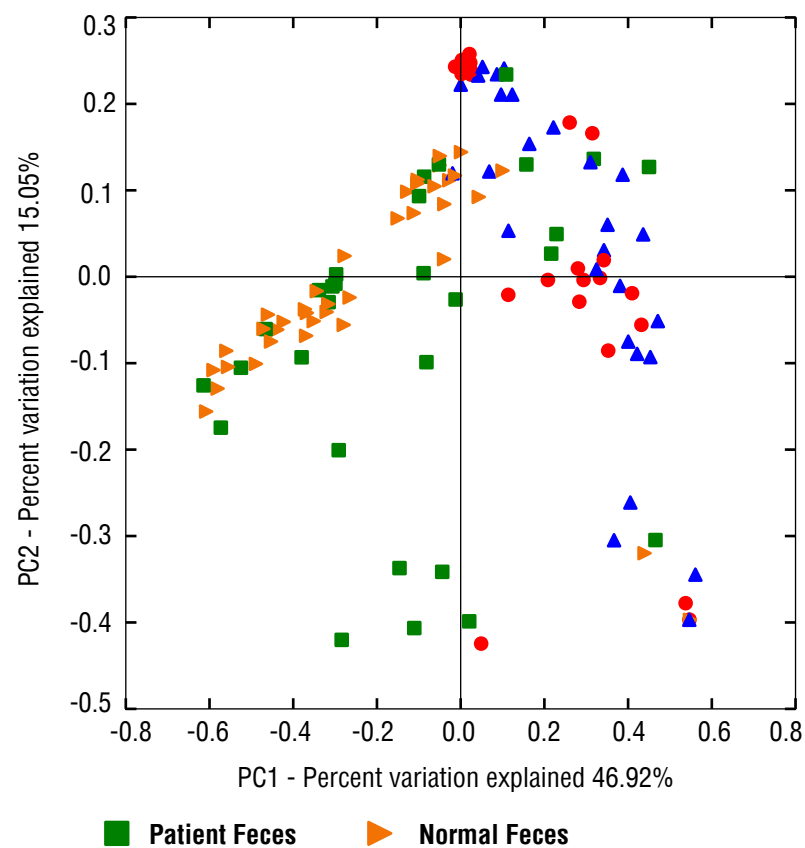

Supplement: Additional file 3: Figure S3 — Sample clustering from the gut and biliary tract based on a Unifrac PCoA analysis of bacterial 16S rRNA-derived OTUs. [file 1471-2164-14-669-S3.pdf]

**Figure S4**

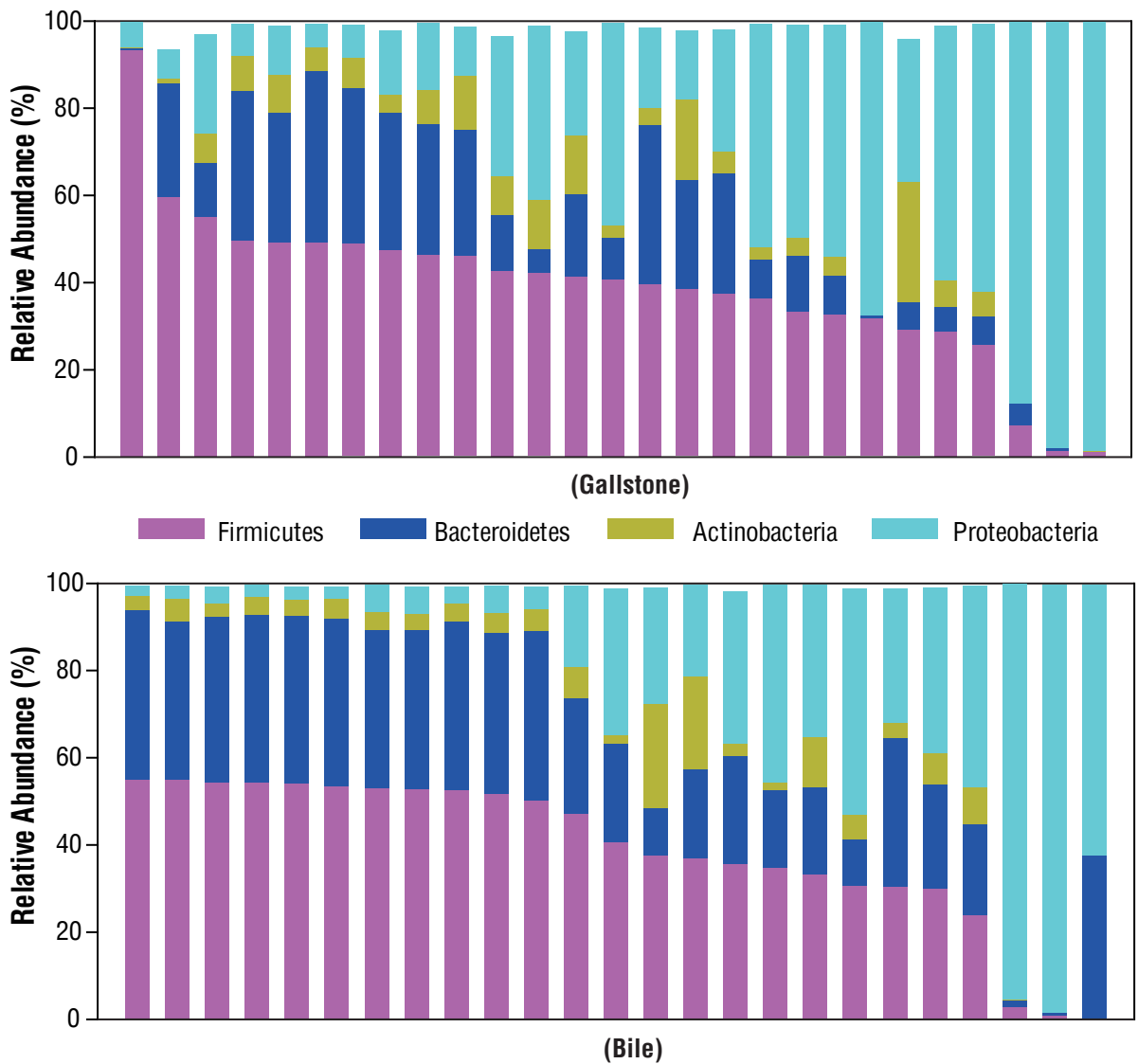

Supplement: Additional file 6: Figure S4 — Inter-subject variations of the top four bacteria phyla present in bile and gallstones. [file 1471-2164-14-669-S6.pdf]
