# Supplementary material for: Gut microbiota dysbiosis and bacterial community assembly associated with cholesterol gallstones in large-scale study
Source: BMC Genomics. 2013 Oct 1;14:669. doi: 10.1186/1471-2164-14-669 (PMC3851472; doi:10.1186/1471-2164-14-669)
Supplement: Additional file 4: Table S1 — Potentially significant bacteria species associated with the presence of gallstones. [file 1471-2164-14-669-S4.doc]

**Table S1 Potential significant bacteria species associated with the presence of gallstone**

| **OTU ID** | **Mean abundance**  **(bile)** | **Mean abundance**  **(Gallstone)** | **Phyla** | **Similar Species** | **Max ID** | **E-value** | **Accession No.**  **in Genbank** |
| --- | --- | --- | --- | --- | --- | --- | --- |
| OTU003 | 0.16% | 0.84% | Actinobacteria | Dietzia schimae strain YIM 65001 | 98% | 7.00E-164 | NR_044482 |
| OTU005 | 0.43% | 0.34% | Actinobacteria | Arthrobacter citreus strain DSM 20133 | 98% | 1.00E-157 | NR_026188 |
| OTU006 | 0.99% | 0.89% | Actinobacteria | Arthrobacter citreus strain DSM 20133 | 98% | 4.00E-161 | NR_026188 |
| OTU007 | 0.43% | 0.39% | Actinobacteria | Rothia mucilaginosa DY-18 | 99% | 8.00E-168 | NC_013715**^#^** |
| OTU008 | 1.58% | 2.38% | Actinobacteria | Propionibacterium acnes C1 | 99% | 2.00E-164 | NC_018707**^#^** |
| OTU009 | 0.20% | 0.36% | Actinobacteria | Propionibacterium acnes C1 | 98% | 3.00E-157 | NC_018707**^#^** |
| OTU021 | 1.18% | 0.70% | Bacteroidetes | Bacteroides vulgatus ATCC 8482 | 99% | 2.00E-158 | NC_009614**^#^** |
| OTU022 | 0.40% | 0.96% | Bacteroidetes | Bacteroides coprophilus DSM 18228 | 98% | 9.00E-168 | NR_041461 |
| OTU023 | 0.72% | 0.46% | Bacteroidetes | Bacteroides stercoris ATCC 43183 | 98% | 1.00E-156 | NR_027196 |
| OTU024 | 0.25% | 1.01% | Bacteroidetes | Bacteroides plebeius DSM 17135 strain M12 | 99% | 1.00E-161 | NR_041277 |
| OTU025 | 1.77% | 0.69% | Bacteroidetes | Bacteroides dorei DSM 17855 strain JCM 13471 | 99% | 1.00E-172 | NR_041351 |
| OTU026 | 0.41% | 0.15% | Bacteroidetes | Bacteroides coprocola DSM 17136 strain M16 | 99% | 2.00E-119 | NR_041278 |
| OTU027 | 0.16% | 0.11% | Bacteroidetes | Bacteroides vulgatus ATCC 8482 | 99% | 6.00E-159 | NC_009614**^#^** |
| OTU028 | 0.34% | 0.43% | Bacteroidetes | Bacteroides caccae strain ATCC 43185 | 97% | 9.00E-153 | NR_026242 |
| OTU037 | 0.32% | 0.22% | Bacteroidetes | Alistipes putredinis strain ATCC 29800 | 97% | 3.00E-152 | NR_025909 |
| OTU039 | 0.67% | 0.60% | Bacteroidetes | Chryseobacterium bovis strain H9 | 98% | 7.00E-164 | NR_044166 |
| OTU042 | 1.04% | 1.82% | Firmicutes | Anoxybacillus flavithermus WK1 | 99% | 1.00E-98 | NC_011567**^#^** |
| OTU043 | 1.36% | 0.88% | Firmicutes | Staphylococcus epidermidis RP62A | 98% | 2.00E-164 | NC_002976**^#^** |
| OTU044 | 1.92% | 2.12% | Firmicutes | Lactococcus lactis subsp. lactis CV56 | 98% | 2.00E-102 | NC_017486**^#^** |
| OTU045 | 4.41% | 4.59% | Firmicutes | Lactococcus raffinolactis strain DSM 20443 | 99% | 1.00E-146 | NR_044359 |
| OTU049 | 0.21% | 0.20% | Firmicutes | Clostridium sp. BNL1100 | 98% | 1.00E-82 | NC_016791**^#^** |
| OTU051 | 0.18% | 0.19% | Firmicutes | Clostridium bartlettii DSM 16795 | 98% | 4.00E-110 | NR_027573 |
| OTU055 | 1.44% | 1.43% | Firmicutes | Clostridium difficile BI1 | 99% | 2.00E-163 | NC_017179**^#^** |
| OTU056 | 0.25% | 0.21% | Firmicutes | Eubacterium rectale ATCC 33656 | 99% | 8.00E-163 | NC_012781**^#^** |
| OTU081 | 0.56% | 0.46% | Firmicutes | Clostridium sp. BNL1100 | 100% | 3.00E-77 | NC_016791**^#^** |
| OTU091 | 0.66% | 0.51% | Firmicutes | Megamonas hypermegale strain DSM 1672 | 97% | 4.00E-157 | NR_025514 |
| OTU092 | 1.74% | 2.19% | Proteobacteria | Caulobacter segnis ATCC 21756 | 99% | 4.00E-104 | NC_014100**^#^** |
| OTU093 | 0.32% | 0.32% | Proteobacteria | Afipia broomeae strain F186 | 99% | 9.00E-153 | NR_029200 |
| OTU094 | 0.16% | 0.15% | Proteobacteria | Dinoroseobacter shibae DFL 12 | 98% | 8.00E-143 | NC_009952**^#^** |
| OTU097 | 0.43% | 0.62% | Proteobacteria | Citrobacter murliniae strain CDC 2970-59 | 99% | 3.00E-168 | NR_028688 |
| OTU098 | 0.90% | 0.85% | Proteobacteria | Escherichia coli str. K-12 substr. MG1655 | 98% | 6.00E-159 | NC_000913**^#^** |
| OTU099 | 0.57% | 0.96% | Proteobacteria | Escherichia coli str. K-12 substr. MG1655 | 98% | 8.00E-163 | NC_000913**^#^** |
| OTU100 | 0.36% | 0.39% | Proteobacteria | Escherichia coli str. K-12 substr. MG1655 | 99% | 6.00E-169 | NC_000913**^#^** |
| OTU102 | 1.20% | 1.32% | Proteobacteria | Acinetobacter calcoaceticus PHEA-2 | 98% | 3.00E-157 | NC_016603**^#^** |
| OTU104 | 0.72% | 0.93% | Proteobacteria | Pseudomonas putida GB-1 | 98% | 6.00E-159 | NC_010322**^#^** |
| OTU106 | 0.12% | 0.33% | Thermi | Meiothermus silvanus DSM 9946 | 99% | 4.00E-161 | NC_014212**^#^** |

**^#^**represents those bacterial species with public genomes
