# Supplementary material for: Gut microbiota dysbiosis and bacterial community assembly associated with cholesterol gallstones in large-scale study
Source: BMC Genomics. 2013 Oct 1;14:669. doi: 10.1186/1471-2164-14-669 (PMC3851472; doi:10.1186/1471-2164-14-669)
Supplement: Additional file 5: Table S2 — Identification of bacterial factors facilitating gallstone formation from 15 similar bacteria species with known genomes. [file 1471-2164-14-669-S5.doc]

**Table S2.** Identification of bacterial factors facilitating gallstone formation from 15 similar bacteria species with known genomes

| **Similar species** | **Genbank Accession** | ***MDR*** | ***BSH*** | ***bG*** | ***phL*** |
| --- | --- | --- | --- | --- | --- |
| ***Acinetobacter calcoaceticus* PHEA-2** | NR_026188 | 21 | 1 | 0 | 2 |
| ***Anoxybacillus flavithermus* WK1** | NC_011567 | 32 | 0 | 0 | 3 |
| ***Bacteroides vulgatus* ATCC 8482** | NC_009614 | 30 | 3 | 24 | 2 |
| ***Clostridium difficile* BI1** | NC_017179 | 57 | 0 | 0 | 4 |
| ***Caulobacter segnis* ATCC 21756** | NC_014100 | 25 | 0 | 2 | 0 |
| ***Clostridium* sp. BNL1100** | NC_016791 | 58 | 0 | 2 | 2 |
| ***Dinoroseobacter shibae* DFL 12** | NC_009952 | 47 | 0 | 0 | 2 |
| ***Escherichia coli* str. K-12 substr. MG1655** | NC_000913 | 40 | 0 | 3 | 4 |
| ***Eubacterium rectale* ATCC 33656** | NC_012781 | 37 | 2 | 3 | 4 |
| ***Lactococcus lactis* subsp. lactis CV56** | NC_017486 | 27 | 2 | 1 | 3 |
| ***Meiothermus silvanus* DSM 9946** | NC_014212 | 43 | 0 | 0 | 0 |
| ***Propionibacterium acnes* C1** | NC_018707 | 34 | 0 | 2 | 2 |
| ***Pseudomonas putida* GB-1** | NC_010322 | 59 | 0 | 0 | 5 |
| ***Rothia mucilaginosa* DY-18** | NC_013715 | 16 | 0 | 0 | 1 |
| ***Staphylococcus epidermidis* RP62A** | NC_002976 | 21 | 1 | 0 | 4 |

*MDR*, multidrug-resistance efflux pump proteins. *BSH*, bile salt hydrolase. *bG*, beta-glucuronidase. *phL*, phospholipase.
